# Trends in paediatric rheumatology referral times and disease activity indices over a ten-year period among children and young people with Juvenile Idiopathic Arthritis: results from the childhood arthritis prospective Study

**DOI:** 10.1093/rheumatology/kew021

**Published:** 2016-03-25

**Authors:** Flora McErlane, Helen E. Foster, Roberto Carrasco, Eileen M. Baildam, S. E. Alice Chieng, Joyce E. Davidson, Yiannis Ioannou, Lucy R. Wedderburn, Wendy Thomson, Kimme L. Hyrich

**Affiliations:** ^1^Paediatric Rheumatology, Great North Children's Hospital, Newcastle Hospitals NHS Foundation Trust,; ^2^Rheumatology, Institute Cellular Medicine, Newcastle University, Newcastle upon Tyne,; ^3^Arthritis Research UK Centre for Epidemiology, Centre for Musculoskeletal Research, Institute for Inflammation and Repair, Faculty of Medical and Human Sciences, University of Manchester, Manchester,; ^4^Paediatric Rheumatology, Alder Hey Children's Hospital, Liverpool,; ^5^Rheumatology, Royal Manchester Children's Hospital, Manchester,; ^6^Paediatric Rheumatology, Royal Hospital for Children, Glasgow,; ^7^Paediatric Rheumatology, Royal Hospital for Sick Children, Edinburgh,; ^8^Arthritis Research UK Centre for Adolescent Rheumatology, Division of Medicine, University College London (UCL),; ^9^Infection, Inflammation and Rheumatology Section, and Arthritis Research UK Centre for Adolescent Rheumatology, UCL Institute of Child Health, London,; ^10^Arthritis Research UK Centre for Genetics and Genomics, Centre for Musculoskeletal Research, Institute for Inflammation and Repair Faculty of Medical and Human Sciences, University of Manchester, and; ^11^NIHR Manchester Musculoskeletal Biomedical Research Unit, Central Manchester NHS Foundation Trust, Manchester Academic Health Science Centre, Manchester, UK

**Keywords:** juvenile idiopathic arthritis, outcomes research, epidemiology, disease activity, treatment

## Abstract

**Objectives.** The medical management of JIA has advanced significantly over the past 10 years. It is not known whether these changes have impacted on outcomes. The aim of this analysis was to identify and describe trends in referral times, treatment times and 1-year outcomes over a 10-year period among children with JIA enrolled in the Childhood Arthritis Prospective Study.

**Methods.** The Childhood Arthritis Prospective Study is a prospective inception cohort of children with new-onset inflammatory arthritis. Analysis included all children recruited in 2001–11 with at least 1 year of follow-up, divided into four groups by year of diagnosis. Median referral time, baseline disease pattern (oligoarticular, polyarticular or systemic onset) and time to first definitive treatment were compared between groups. Where possible, clinical juvenile arthritis disease activity score (cJADAS) cut-offs were applied at 1 year.

**Results.** One thousand and sixty-six children were included in the analysis. The median time from symptom onset and referral to first paediatric rheumatology appointment (22.7–24.7 and 3.4–4.7 weeks, respectively) did not vary significantly (∼20% seen within 10 weeks of onset and ∼50% within 4 weeks of referral). For oligoarticular and polyarticular disease, 33.8–47 and 25.4–34.9%, respectively, achieved inactive disease by 1 year, with ∼30% in high disease activity at 1 year. A positive trend towards earlier definitive treatment reached significance in oligoarticular and polyarticular pattern disease.

**Conclusion.** Children with new-onset JIA have a persistent delay in access to paediatric rheumatology care, with one-third in high disease activity at 1 year and no significant improvement over the past 10 years. Contributing factors may include service pressures and poor awareness. Further research is necessary to gain a better understanding and improve important clinical outcomes.

Rheumatology key messages

- Children with JIA continue to have a protracted interval between initial presentation and specialist paediatric rheumatology care.
- Approximately one-third of children with JIA remain in high disease activity 1 year after presentation.
- There has been no significant change in 1-year outcomes in JIA over the past 10 years.

## Introduction

Inflammatory arthritis occurs in ∼10:100 000 children each year [1], with the majority subsequently diagnosed with JIA. JIA is an umbrella term, summarizing the ILAR classification system for the markedly heterogeneous group of chronic childhood-onset arthritides [2, 3]. Delay in access to paediatric rheumatology care is important and predicts poorer disease outcomes for children and young people (CYP) with JIA [4–6]. The UK British Society for Paediatric and Adolescent Rhuematology (BSPAR) Standards of Care for CYP with JIA (2009) are evidence- and consensus-derived standards outlining the minimal level of care for CYP with JIA. The standards stipulate that all children with JIA should be assessed by a paediatric rheumatology team within 10 weeks of symptom onset and within 4 weeks of referral [7]. In a recent (2013) study, 10 UK paediatric rheumatology centres participated in a retrospective review of clinical practice; 41% patients (175/428) were seen within 10 weeks of symptom onset and 60% (186/311) had the first paediatric rheumatology appointment within 4 weeks of the initial referral [8]. However, there are no prospective UK-wide studies of trends in access to care and associated clinical outcomes.

The advent of new biologic treatment agents and the growing evidence base for the treatment of JIA have resulted in a new era in the management of JIA, with an expectation that early aggressive therapy will improve remission rates, prevent damage and normalize functional outcomes [9]. Early aggressive treatment of children with polyarticular JIA enrolled in the Trial of Early Aggressive Therapy clinical study was associated with low disease activity and prolonged periods of clinically inactive disease during a 2-year extension study [10]. It is not yet known whether these recent changes in our understanding of the medical management of JIA have impacted on prescribing patterns and outcomes in routine clinical practice. Recent intensification of early treatment regimens may further compound the impact of time to diagnosis on disease outcomes.

The assessment of disease activity in JIA has recently been simplified with the development of the juvenile arthritis DAS (JADAS), a four-variable composite disease activity score specific to JIA [11]. The JADAS3, also referred to as cJADAS, is a more feasible three-variable clinical tool, which does not include an acute phase reactant [12]. To aid interpretation of scores, cut-off values corresponding to a number of disease states have been validated for both composite indices [13–15]. The cut-offs have not yet been trialled in routine clinical practice.

This analysis aims to describe trends in referral times, baseline disease severity, time to initial treatment and 1-year outcomes, including the JADAS3, over a 10-year period among children with JIA enrolled in the Childhood Arthritis Prospective Study (CAPS). Identifying trends in access to care and medication is key to understanding the impact of recent research into early aggressive therapy on routine clinical practice.

## Methods

### Study population

Children in this analysis were participants in the CAPS, an ongoing UK prospective inception cohort study launched in 2001 [16, 17]. The aim of the CAPS is to provide long-term outcome data on CYP with new-onset inflammatory arthritis receiving routine specialist care in the UK. Children aged <16 years with a new diagnosis of inflammatory arthritis present for at least 2 weeks, presenting to one of seven UK paediatric rheumatology centres (Liverpool, Manchester, Newcastle, Glasgow, Edinburgh, Great Ormond Street and University College London) are invited to participate. Exclusion criteria are septic arthritis and arthritis related to malignancy, trauma or connective tissue disease (SLE, JDM or MCTD). The CAPS was approved by the UK Northwest Multicentre Research Ethics Committee. Written informed consent has been obtained from the parent(s)/guardians of all participating children, and all able children have provided written assent for the CAPS. This analysis did not require any additional ethical approval as it was a secondary analysis of the anonymized dataset.

### Data collection

Data for this analysis have been collated from the CAPS database and include information from medical records and interview with the child/family, as described previously [16]. At the first appointment, ILAR designation of JIA subtype and core outcome variables (COVs) are documented by the paediatric rheumatologist, and the parent/child is asked to complete a Childhood Health Assessment Questionnaire (CHAQ), including a 10-cm parent global visual analog scale and 10-cm pain visual analog scale. A paediatric rheumatology research nurse interviews the parent(s) and child within 3 months of the initial visit and extracts demographic and clinical data from the medical records. Data are collected at baseline, 6 months and then annually to 5 years, including a confirmation of the underlying diagnosis and ILAR subtype. Date of disease onset is obtained from the family interview or the medical case notes. Date of referral and date of first paediatric rheumatology appointment are extracted from the medical case notes.

### Analysis

All children with a confirmed physician’s diagnosis of JIA recruited between 2001 and 2011 with at least 1 year of follow-up within the study were included in this analysis. The cohort was analysed in four groups of approximately equal size, divided by year of first presentation to paediatric rheumatology (2001–04, 2005–06, 2007–08 and 2009–11). At baseline, median referral times, disease pattern (oligoarticular pattern, polyarticular pattern or systemic onset based on maximal active joint count during the first year and ILAR subtype at 1 year), disease activity defined using the JADAS71 [11] and JADAS3-71 [12] and outcome indices (including active joint count, limited joint count, physician global assessment, parent global evaluation, ESR, CHAQ and pain assessment) were determined for each group. All patients with systemic onset JIA were allocated to the systemic pattern group, regardless of joint involvement. Patients with non-systemic JIA were allocated to the oligoarticular or polyarticular group according to the cumulative joint involvement during the first year of observation.

Time to first anti-rheumatic treatment (excluding NSAIDs) was determined for all children (typically first intra-articular steroid injection for oligoarticular pattern and MTX for polyarticular and systemic pattern). At 1 year, the active joint count, JADAS and JADAS3 were determined for all children with sufficient data available. The cut-off values for JADAS3 (cJADAS) were applied to determine the proportion of children with oligoarticular and polyarticular disease patterns in high disease activity (HDA), moderate disease activity, low disease activity (LDA) and inactive disease at 1 year. The cut-offs cannot be applied to children with systemic onset disease.

Values in each category across the four groups were compared using linear (for continuous variables) and logistic (for binary variables) regression, with year group as the independent variable, adjusting for paediatric rheumatology centre and disease pattern. Time to first definitive treatment was determined within each disease pattern. All analyses were performed using Stata version 11.0 (StataCorp, College Station, TX, USA).

## Results

### Study population

**Fig. 1:**
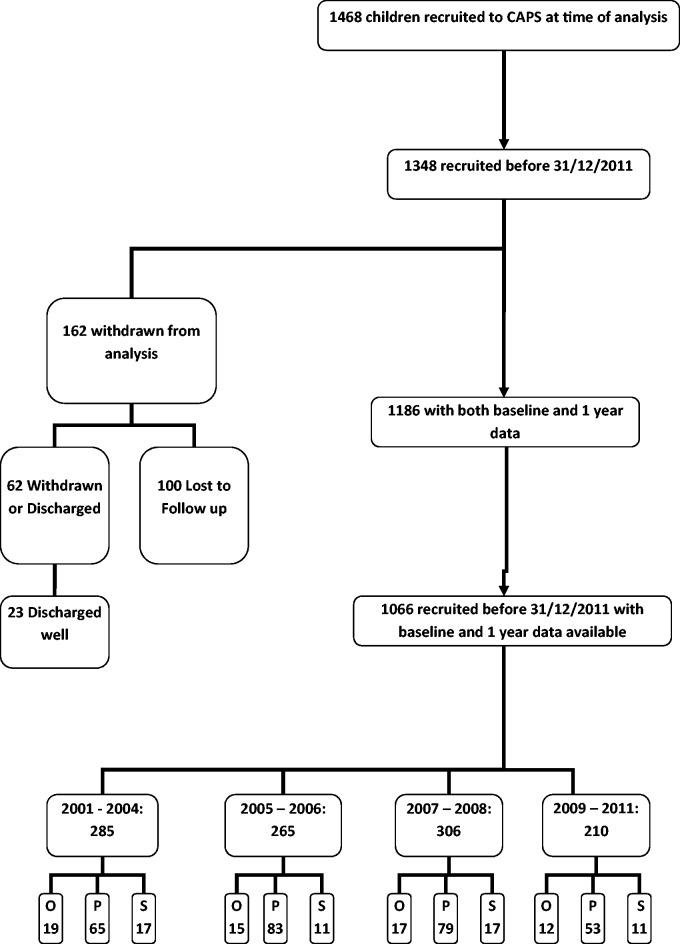
Flow chart for subject inclusion O: oligoarticular pattern JIA; P: polyarticular pattern JIA; S: systemic onset JIA.

In total, 1066 children with baseline and 1-year data available were divided into four groups of approximately equal size by year of diagnosis and included in the primary analysis (Fig. 1). Disease pattern was not available for 79/1066 (7%) children; therefore, the secondary analysis, by disease pattern, included 987 children [comprising oligoarticular pattern disease (651), polyarticular pattern disease (280) and systemic onset disease (56), according to joint counts and ILAR subtype].

### Baseline characteristics, waiting times and referral patterns

The baseline characteristics of the four groups are summarized in Table 1, with no significant difference in age, sex, ethnicity or baseline disease activity measures across the four subgroups.

**Table 1:** Baseline demographics and disease activity measures by disease pattern

Demographics and disease activity indices	Whole cohort	2001–04	2005–06	2007–08	2009–11	P-value^a^
Total, n	1066	285	265	306	210	-
Age, median (IQR), years	7.74 (3.46–11.8)	7.8 (4–11.7)	8.2 (3.5–11.9)	8.76 (3.6–11.9)	6.34 (2.7–11.4)	0.1
Female, n (%)	698 (65)	186 (65)	174 (65.4)	198 (64.5)	140 (66.7)	0.72
Ethnicity, n (%), White, Other	960 (91)	263 (92.3)	249 (94)	274 (89.5)	174 (82.9)	0.07
	106 (9.9)	22 (7.7)	16 (6)	32 (10.5)	36 (17.1)	
Active Joint Count, median (IQR)	2 (1–5)	2 (1–4)	2 (1–6)	2 (1–5)	2 (1–5)	0.82
Limited Joint Count, median (IQR)	1 (1–3)	1 (1–3)	1 (1–4)	1 (0–3)	1 (1–3)	0.29
Physician GA, median (IQR)	29 (16–51)	34.3 (19–60)	33 (20–53)	25 (14–45.5)	25.5 (13–45)	0.03
Parent GE, median (IQR)	21 (5–49)	20 (5–50)	24.5 (8–46.5)	21 (4–51)	25 (12–40)	0.95
ESR, median (IQR)	21 (7–50)	15 (6–44)	26 (7–52)	21 (7.5–51.5)	23 (5–49)	0.08
Pain, median (IQR)	30 (8–59)	36.5 (10–65)	36.5 (12–60)	27 (6–54)	29.5 (9.5–52.5)	0.048
CHAQ, median (IQR)	0.6 (0.1–1.4)	0.8 (0.1–1.5)	0.9 (0.3–1.5)	0.6 (0.1–1.4)	0.6 (0.1–1.3)	0.045
JADAS, median (IQR), n	10.75 (5.7–17.6), 424	10.8 (7–16.2), 135	12.1 (7.2–20.2), 113	9.3 (4.4–18.4), 103	10.4 (4–15.6), 73	0.60
JADAS3-71, median (IQR), n	9.3 (5–14.4), 553	9.4 (5.7–13.6), 190	10 (6.4–16.3), 148	8.6 (4.1–4.2), 128	8.6 (4–14.6), 87	0.35

^a^P-values compare time groups for each variable and are adjusted by hospital and disease pattern. CHAQ: Childhood Health Assessment Questionnaire; GA: global assessment; GE: global evaluation; IQR: interquartile range; JDAS: juvenile arthritis disease activity score.

The median time from both symptom onset and date of referral to first paediatric rheumatology appointment (ranging from 22.7 to 24.7 and from 3.4 to 4.7 weeks, respectively) did not vary significantly across the four subgroup study period (Table 2). Only ∼20% were seen within 10 weeks of symptom onset and ∼50% within 4 weeks of referral, with a significant trend towards longer waiting times following referral for first appointments in more recent years. CYP with systemic onset JIA had significantly shorter waiting times across the 10-year period of the study. The majority of the cohort was referred by secondary care sources [commonly, paediatrics (40%) and orthopaedics (25%)], with a significant increase in the proportion of CYP with oligoarticular and polyarticular patterns referred by paediatricians over the 10-year period of the study (Table 3).

**Table 2:** Referral times by disease pattern

Disease pattern	Whole cohort	2001–04	2005–06	2007–08	2009–11	P-value
Time between symptom onset and first paediatric rheumatology appointment						
All disease patterns, median (IQR), weeks	23.6 (12.3–50.4)	22.7 (11.9–40.1)	23.5 (12.1–52.7)	24.7 (12–58.2)	23.1 (13.2–50.1)	0.28
n = 1066						
Oligoarticular pattern, median (IQR), weeks	23.4 (12.5–50)	22 (12.2–41.6)	22.5 (11.8–46.6)	27.1 (13.6–63.3)	22.9 (13.7–49.6)	0.7
n = 651						
Polyarticular pattern, median (IQR), weeks	26.3 (14.7–51.7)	26.7 (12.8–107)	30.6 (15.5–64.3)	23.7 (13–41.6)	24.9 (14.7–32.7)	0.005
n = 280						
Systemic pattern, median (IQR), weeks	9 (4.3–24.6)	12 (4.7–24)	9.4 (3.7–44.3)	8.4 (3–25.3)	9.3 (5.7–20.8)	0.5
n = 56						
Percentage seen within 10 weeks of symptom onset	19.8	20.5	20.75	19.8	18.7	0.66
Time between referral and first paediatric rheumatology appointment						
All disease patterns, median (IQR), weeks	4	3.4	4	4.7	4.3	0.61
	(1.3–8)	(1.2–7.9)	(1.4–7.3)	(1.4–8)	(1.6–8.7)	
Oligoarticular pattern, median (IQR), weeks	4.3	3.6	3.9	6	4.4	0.9
	(1.9–8.3)	(1.3–8.4)	(1.6–7)	(2–8.3)	(2.1–9.4)	
Polyarticular pattern, median (IQR), weeks	4.4	3.4	5	4.1	5	0.6
	(1.7–8)	(1.4–7.1)	(2.1–8.4)	(1.4–7.6)	(2–7.4)	
Systemic pattern, median (IQR), weeks	0.4	0.4	0.3	0.4	0.4	0.4
	(0–1.4)	(0–1.4)	(0–0.6)	(0–1.4)	(0.3–1.4)	
Percentage seen within 4 weeks of referral	52.9	58.1	55.2	49	50	0.02

IQR: interquartile range.

**Table 3:** Referral sources

Referral sources at presentation by disease pattern	Whole cohort	2001–04	2005–06	2007–08	2009–11	P-value
Oligoarticular pattern (%)						
Primary care (GP)	114 (17.6)	34 (17.5)	24 (15.4)	36 (20.6)	20 (16.4)	0.7
Accident and emergency doctor	51 (7.9)	21 (10.8)	6 (3.8)	14 (8)	10 (8.2)	0.5
Paediatrician	224 (34.6)	48 (24.7)	64 (41)	68 (38.8)	44 (36)	0.02
Orthopaedics	208 (32.1)	81 (41.7)	49 (31.4)	46 (26.3)	32 (26.2)	0.001
Physiotherapist	8 (1.24)	1 (0.5)	2 (1.3)	4 (2.3)	1 (0.8)	0.45
Other	42 (6.5)	9 (4.6)	11 (7)	7 (4)	15 (12.3)	0.048
Polyarticular pattern (%)						
Primary care (GP)	62 (22.6)	22 (35.5)	14 (17.5)	17 (21.5)	9 (17)	0.06
Accident and emergency doctor	15 (5.5)	7 (11.3)	3 (3.75)	4 (5)	1 (1.9)	0.06
Paediatrician	134 (48.9)	13 (21)	48 (60)	39 (49.4)	34 (64.1)	<0.001
Orthopaedics	38 (13.8)	14 (22.6)	8 (10)	12 (15.2)	4 (7.5)	0.08
Physiotherapist	2 (0.7)	0	2 (2.5)	0	0	0.55
Other	23 (8.4)	6 (9.7)	5 (6.25)	7 (8.9)	5 (9.4)	0.8
Systemic onset pattern (%)						
Primary care (GP)	3 (5.4)	2 (11.7)	0	1 (6.25)	0	0.25
Accident and emergency doctor	4 (7.3)	2 (11.7)	1 (9.1)	1 (6.25)	0	0.23
Paediatrician	37 (67.3)	10 (58.8)	9 (81.8)	10 (62.5)	8 (72.7)	0.7
Orthopaedics	4 (7.3)	1 (5.9)	0	2 (12.5)	1 (9.1)	0.5
Physiotherapist	0	0	0	0	0	0
Other	7 (17.3)	2 (11.7)	1 (9.1)	2 (12.5)	2 (18.2)	0.44

GP: general practitioner.

### Disease activity at presentation and 1 year

Disease activity had decreased at 1 year in all disease patterns (Table 4). It was not possible to calculate JADAS71 or JADAS3-71 at 1 year for all children, with complete data for all four items in JADAS71 available in 266/987 (27%) patients and complete data for all three items in JADAS3-71 available in 546/987 (55%) patients. The differences between the cohorts with and without all variables available to calculate JADAS have been described in detail in a previous article [12]. In particular, CYP with JADAS and cJADAS scores had significantly higher physician global scores than the remainder of the cohort. The JADAS3 or cJADAS cut-off criteria were applied to those children with sufficient data available to calculate at 1 year (JADAS oligoarthritis: inactive disease ⩽1, MDA ⩽2 and HDA >4.2; JADAS polyarthritis: inactive disease ⩽1, MDA ⩽3.8 and HDA >10.5; cJADAS oligoarthritis inactive disease ⩽1, LDA ⩽1.5, MDA 1.51–4 and HDA >4; and cJADAS polyarthritis inactive disease ⩽1, LDA ⩽2.5, MDA 2.51–8.5 and HDA >8.5/10.5) [13–15]. Between 33.8 and 47% of children with oligoarticular disease pattern and JADAS3 available had achieved inactive disease by 1 year, and this dropped to between 25.4 and 34.9% in polyarticular disease. Around 30% children with JADAS3 available remained in HDA at 1 year.

**Table 4:** Disease activity at 1 year by disease pattern

Disease activity measure	1 year				
	2001–04	2005–06	2007–08	2009–11	P-value
Oligoarticular disease pattern					
AJC, median (IQR)	0 (0–1)	0 (0–1)	0 (0–1)	0 (0–0)	0.45
JADAS71, median (IQR), n	5.1 (2.9–9.1), 33	4.95 (1.45–7.6), 30	3.2 (0.6–5.6), 33	4 (2–11), 32	0.006
JADAS3-71, median (IQR), n	2 (0.4–4.7), 111	2.1 (0.2–5.7), 91	1.5 (0.2–4.5), 83	2.15 (0.5–5.2), 62	0.001
Percentage with inactive disease	39.65	42.8	47	33.8	0.79
Percentage with LDA	46	47.25	53	43.55	0.9
Percentage with ModDA	23.43	20.8	20.5	22.6	0.81
Percentage with HAD	30.6	32	26.5	33.8	0.9
Polyarticular disease pattern					
AJC, median (IQR)	0 (0–5)	0 (0–4)	0 (0–2)	0 (0–2)	0.1
JADAS3-71, median (IQR), n	5.8 (1–12.1), 47	2.8 (0.9–8), 48	2.8 (0.3–6.2), 40	2.1 (0.7–13), 29	0.71
Percentage with inactive disease	25.4	29.1	34.9	34.5	0.27
Percentage with LDA	33.9	50.1	47.5	51.8	0.1
Percentage with ModDA	32	33.4	37.3	17.2	0.27
Percentage with HAD	34.1	16.6	14.9	31.1	0.46

AJC: active joint count; HDA: high disease activity; IQR: interquartile range; JDAS: juvenile arthritis disease activity score; LDA: low disease activity; ModDA: moderate disease activity.

### Time to first anti-rheumatic treatment

Following initial assessment by a paediatric rheumatologist, there was a positive trend towards earlier definitive treatment, reaching significance in oligoarticular and polyarticular pattern disease (Table 5). However, there was no significant difference in the proportion of patients receiving biologic therapies during the first year.

**Table 5:** Time to first definitive treatment by disease pattern

Time to first definitive treatment by disease pattern	2001–04	2005–06	2007–08	2009–11	P-value
Oligoarticular disease pattern					
Total, n (%)	196 (68.8)	158 (60)	175 (57.2)	122 (58.1)	0.25
No. of patients receiving IA steroid ever in first year (%)	114	121	139	97	0.0001
	(58.2)	(76.1)	(79)	(79.5)	
Median days from first PRh to first IA steroid (IQR)	25.5	25	19	19	0.04
	(9–65)	(7–49)	(8–48)	(9–48)	
No. of patients receiving biologic agents ever in first year (%)	5	4	6	3	0.9
	(2.55)	(2.53)	(3.41)	(2.46)	
Polyarticular disease pattern					
n (%)	65 (22.8)	83 (31.3)	79 (25.8)	53 (25.2)	0.5
MTX ever in first year, n (%)	55 (84.6)	72 (86.8)	76 (96.2)	49 (92.5)	0.081
Median days from first PRh to first MTX (IQR)	27 (1–79)	17 (1–43)	5 (0–17)	11 (0–84)	0.03
Median days from first PRh to first oral/i.v./i.m. steroid (IQR)	14 (1–140)	0 (0–21)	9 (3–41)	13 (6–68)	0.63
No. of patients receiving biologic agents ever in first year (%)	4 (6.15)	16 (19.3)	17 (21.25)	10 (18.9)	0.06
Systemic disease pattern					
n (%)	17 (6)	11 (4.2)	17 (5.5)	11 (5.2)	0.40
MTX ever in first year, n (%)	15 (88.2)	11 (100)	156 (88.3)	89 (72.8)	0.66
Median days from first PRh to first MTX (IQR)	37 (14–78)	14 (9–25)	15 (6–24)	15 (13–26)	0.07
Median days from first PRh to first oral/i.v./i.m. steroid (IQR)	53 (4–117)	8 (6–20)	7.5 (5–31)	12.5 (2–19)	0.07
No. of patients receiving biologic agents ever in first year (%)	3 (17.65)	2 (18.2)	2 (11.8)	1 (9.1)	0.5

IA: intra-articular; IQR: interquartile range; PRh: paediatric rheumatology.

## Discussion

This study describes trends in referral time and time to first definitive treatment and trends in disease-related outcomes at 1 year in a large real-world data set of children with all ILAR subtypes of JIA collected over a contemporaneous 10-year time period. The majority of CYP in this study had a protracted interval between initial presentation and specialist paediatric rheumatology care, with no significant change in the time from symptom onset to first paediatric rheumatology appointment over the 10-year study period. A minority (20%) of the cohort was seen within 10 weeks of symptom onset, with no significant change following the publication of the BSPAR Standards of Care document in 2009. Children with systemic onset JIA had the shortest interval between initial symptom and first paediatric rheumatology appointment, and this is not surprising given that these children are often systemically unwell and likely to present early to paediatric services, with rapid assessment and referral. It is interesting that children with oligoarticular and polyarticular presentations had a similar duration of delay in access to care. The presentation of arthritis can be subtle in children, with early morning stiffness and joint restriction more prominent than pain, and function frequently well preserved. Diagnosis therefore requires a high index of suspicion and good musculoskeletal examination skills. Access to care is a complex issue [6], with multifactorial influences over health-seeking behaviour, availability of services and recognition of JIA by health-care professionals. Routes of referral to paediatric rheumatology care are often complex, involving primary and secondary care; it is known that many doctors to whom CYP may present lack self-reported confidence in their musculoskeletal clinical examination skills [18] and, indeed, many doctors in primary care have not had any training in paediatrics [19].

A number of other factors may influence access to definitive care. We have previously described an association between normal ESR and longer duration of symptoms at presentation [16]. In the same analysis, there was no significant difference in age at presentation, baseline CHAQ, physician’s global assessment, parent’s general evaluation or pain scores between children with longer (>4 months) or shorter disease duration at presentation. Those with a longer duration of symptoms had higher active and limited joint counts, but there was no difference in the frequencies of upper or lower limb involvement.

It is important to note that the accurate capture of disease onset data can be challenging. In this study, disease onset was assumed to be the earliest onset date recorded in either the medical case notes or interview with the family. The additional time for reflection prior to interview, following the initial paediatric rheumatology consultation, may result in more accurate onset data.

The wide variation in referral sources over the 10-year period of this study demonstrates the complexity of pathways to care in JIA. Two-thirds of the children in this study were referred to paediatric rheumatology by secondary care services, such as paediatric and orthopaedic teams, rather than the initial point of medical contact (frequently primary care). This important observation has been highlighted previously in the CAPS cohort, including an analysis of the impact of referral source on symptom duration at first paediatric rheumatology appointment [16]. There is a significant difference in symptom duration between referral sources, with the longest delay occurring in children referred from routes other than general or musculoskeletal care (including plastic surgery, ophthalmology, otolaryngology, neurology, physiotherapy, adult rheumatology and direct parent referral). A similar variation in referral pathways and interval to first paediatric rheumatology assessment has been reported in other cohorts, and variable awareness of JIA amongst health-care professionals is likely to be important [20].

This observed delay in access to care mirrors the published literature [9, 17, 20] and is likely to have an adverse impact on long-term clinical outcomes [21]. It is disappointing that there has been no significant improvement in access to care over the past 10 years, particularly in view of the publication of the BSPAR Standards of Care in 2009. Since the emergence of the Standards of Care, there have been considerable efforts to raise awareness of JIA. Educational strategies to improve musculoskeletal clinical skills of all doctors who may come into contact with CYP begin with medical students and the teaching of paediatric gait arms legs spine (pGALS) [22], which is now taught at many medical schools (K. Baker British Society for Rheumatology, unpublished results), through to up-skilling paediatricians (including musculoskeletal clinical skills in professional examinations since 2009) and supporting primary care though educational events (e.g. British Medical Journal Masterclass, Primary Care Rheumatology Society), e-resources (e.g. Arthritis Research UK website) and e-learning (paediatric musculoskeletal matters, www.pmmonline.org, launched 2014). Guidelines and e-resources (such as National Health Service Map of Medicine, E-Learning for Health) now include reference to JIA and encourage primary care physicians and paediatricians to consider JIA in the diagnostic pathways of limp, limb pain and joint swelling. Empowering families to seek health care is also needed, and it is known that teachers play an important role in early recognition of JIA (T. Rapley, C.R. May, H.E.F., unpublished results). Further targeted education for schools, nursery workers and health visitors is likely to be helpful. Clinical networks improve equity of access to care, delivered as close to home as possible, and may improve local awareness of JIA, particularly if delivered in conjunction with an education programme [24].

The proportion of children seen within 4 weeks of initial referral decreased significantly over the 10-year period of the study, from 58.1 to 49%. Increased clinic waiting times reflect the increased service pressures on tertiary paediatric rheumatology centres over the past 10 years and further highlight the need for improved care pathways within the specialty.

For the purposes of this study, the first anti-rheumatic treatment for children with oligoarticular pattern JIA was defined as first intra-articular corticosteroid (IA steroid) injection, and the first disease-modifying treatment for children with polyarticular and systemic onset JIA was defined as first MTX (oral or subcutaneous), although we acknowledge the importance of both treatment modalities for all subtypes. Once within the paediatric rheumatology service, there was a decrease in the median time to first definitive treatment across all subtypes, although small numbers prevented robust comparisons among children with systemic onset disease. While the improvement is reassuring, a median delay of almost 3 weeks was observed, which may be explained in part by time waiting for general anaesthetic (intra-articular injections) or the time required to counsel patients, organize prescription and delivery of MTX and allow varicella immunization if indicated.

The decrease in time to first definitive treatment paralleled a trend towards higher numbers of CYP with polyarticular disease patterns achieving inactive disease at 1 year in the later years of the study. However, the proportion of children prescribed biologic therapies did not change significantly over the 10-year study period.

Overall, ∼30% of CYP remained in HDA at 1 year despite an increasing choice of therapies. Shorter disease duration at presentation predicts higher likelihood of attaining and maintaining clinically inactive disease [23]. Improving access to definitive care may therefore be one way to improve short- to medium-term outcomes in JIA. Early aggressive therapy has been shown to result in relatively high numbers of children with polyarticular JIA achieving clinically inactive disease by 6 months [24]. It is perhaps disappointing that there was no significant difference over the 10 years in the proportion of patients receiving biologic therapies during the first year. There were no national treatment guidelines available during the 10-year study period, and children were treated according to the local clinician’s discretion. National treatment guidelines, perhaps incorporating targeted treatment regimens, might improve clinical outcomes. Treating to target in JIA will not require novel medications, but by aggressively chasing predefined disease activity targets, treatment regimens will be intensified and outcomes may improve.

Accurate comparison with outcomes reported by other prospective cohort studies is challenging because of variation in disease definition, outcome definitions and statistical methods. In 2015, the probability of attaining inactive disease by 1 year (2005–10) was reported as 44.9% in the ReACCh-Out cohort [25]. In 2012, 77% of 149 patients achieved their first episode of inactive disease by 2 years [26]. Variation in the definition of inactive disease means that neither study can be compared directly with rates of inactive disease in the present cohort (defined by the JADAS cut-offs).

The strengths of the present study lie in detailed clinical information across a large number of children with a relatively rare disease collected over a very long period of time. However, these types of data are not without their limitations, common to all observational studies within a real-world clinical setting. One hundred and sixty-two children were lost to follow-up early in the study and therefore excluded. An additional proportion of children had missing data, making it difficult to calculate JADAS3-71 in all children. Missing data included the ESR and the physician global and parent global assessments, as described in a previous report [12]. Children with sufficient data available to calculate the JADAS71 and JADAS3-71 had significantly higher physician global scores than those without. This implies that the true proportion of children with inactive disease by 1 year may be higher than reported.

Collection of COV data should be integral to routine clinical care to ensure that important targets of clinical remission are achieved in a timely fashion. With this in mind, the Standards of Care stipulate that all CYP with JIA should have the COVs measured at each clinical review. The COVs were developed to standardize the assessment of therapeutic response in clinical trials involving children with JIA [27]. Although they remain the gold-standard assessment tool in the context of clinical trials, the collection of the COVs is not always feasible in the clinical setting. For example, the ESR is not routinely measured in all children with JIA, particularly those with oligoarticular pattern disease, and this is why the JADAS3 is so important in the clinical setting. One potential solution would be the development of a minimal clinical data set, designed to be both feasible and useful in the clinical setting.

For the purposes of this descriptive analysis, the cohort was subdivided into four groups of approximately equal size by year of diagnosis (2001–04, 2005–06, 2007–08 and 2009–11). The four groups were recruited over variable time periods (ranging from 2 to 4 years), and there may have been some variation in the management of children recruited at the start and children recruited towards the end of the longer time periods.

Children in this study were subdivided into oligoarticular, polyarticular and systemic onset disease pattern, according to joint count data. However, it is widely recognized that children with oligoarticular JIA may have less aggressive disease than children with psoriatic JIA or enthesitis-related JIA and an oligoarticular disease course. Children with oligoarticular JIA may have quite different outcomes from the oligoarticular disease pattern group as a whole. The classification of children into oligoarticular, polyarticular and systemic onset disease patterns may be an important limitation of the present study, reflecting the challenges of the ILAR classification system in the clinical context.

### Conclusion

Despite guidelines emphasizing early assessment by paediatric rheumatology clinicians, approximately half of the children with new-onset JIA were not seen within 4 weeks of referral, with only 20% within 10 weeks of symptom onset. The reasons for the former finding may be related to service pressures, with the latter multifactorial, relating to both public and physician education. However, it is encouraging to see more rapid introduction of treatment and associated improvements in outcome. Further research is necessary to understand why approximately one-third of children continue to have active disease at 1 year. This study further highlights a significant and sustained delay in referral to paediatric rheumatology and the need for greater effort to facilitate early recognition and triage by health-care professionals who may have the initial contact with musculoskeletal presentations in CYP. Delayed or inequitable access to tertiary care may impact on outcomes and is therefore is a priority to identify and address.
